# Locally applied heat stress during exercise training may promote adaptations to mitochondrial enzyme activities in skeletal muscle

**DOI:** 10.1007/s00424-024-02939-8

**Published:** 2024-03-06

**Authors:** Ed Maunder, Andrew King, Jeffrey A. Rothschild, Matthew J. Brick, Warren B. Leigh, Christopher P. Hedges, Troy L. Merry, Andrew E. Kilding

**Affiliations:** 1 Sports Performance Research Institute New Zealand, Auckland University of Technology, Auckland, New Zealand; 2 Orthosports North Harbour, AUT Millennium, Auckland, New Zealand; 3 Discipline of Nutrition, School of Medical Sciences, University of Auckland, Auckland, New Zealand

**Keywords:** Heat, Mitochondria, Exercise, Muscle

## Abstract

There is some evidence for temperature-dependent stimulation of mitochondrial biogenesis; however, the role of elevated muscle temperature during exercise in mitochondrial adaptation to training has not been studied in humans in vivo. The purpose of this study was to determine the role of elevating muscle temperature during exercise in temperate conditions through the application of mild, local heat stress on mitochondrial adaptations to endurance training. Eight endurance-trained males undertook 3 weeks of supervised cycling training, during which mild (~ 40 °C) heat stress was applied locally to the upper-leg musculature of one leg during all training sessions (HEAT), with the contralateral leg serving as the non-heated, exercising control (CON). *Vastus lateralis* microbiopsies were obtained from both legs before and after the training period. Training-induced increases in complex I (fold-change, 1.24 ± 0.33 vs. 1.01 ± 0.49, *P* = 0.029) and II (fold-change, 1.24 ± 0.33 vs. 1.01 ± 0.49, *P* = 0.029) activities were significantly larger in HEAT than CON. No significant effects of training, or interactions between local heat stress application and training, were observed for complex I–V or HSP70 protein expressions. Our data provides partial evidence to support the hypothesis that elevating local muscle temperature during exercise augments training-induced adaptations to mitochondrial enzyme activity.

## Introduction

Exercise training can increase mitochondrial volume density, oxidative capacity, and enzyme content and activity [8, 15, 39]. Physiological stresses induced by exercise transiently increase the expression of proteins coordinating these mitochondrial adaptations, via activation of existing proteins and mRNA expression [14]. Exercise stresses thought to influence signalling related to mitochondrial adaptations include cytosolic adenosine monophosphate (AMP) and Ca^2+^ accumulation, oxidative stress, and substrate depletion [18, 30, 32, 36, 44]. Increased muscle temperature is a ubiquitous exercise stress [6], but the role of transient exercise-induced elevations in muscle temperature on mitochondrial adaptation in response to chronic endurance training is not known [12].

Temperature-dependent stimulation of mitochondrial biogenesis has been observed in in vitro models [23], animal models of post-exercise passive heat stress [41], and following repeated bouts of passive locally-applied heat stress in humans [10]. In endurance-trained males, we recently reported 3 weeks of endurance training performed under moderate whole-body heat stress (33 °C) increased maximal vastus lateralis citrate synthase activity, whereas matched training in 18 °C did not [28]. It is possible greater muscle temperature during training sessions in 33 °C contributed to the observed adaptive effects [3]. Increased muscle temperature during exercise increases glycogenolysis [5, 40], which may increase 5′AMP-activated protein kinase (AMPK) activation through reduced inhibition of AMPK via the glycogen-binding domain [17, 30]. Similarly, heat stress has been shown to upregulate heat shock protein 70 (HSP70) expression [10, 41], which promotes mitochondrial biogenesis [13] via the role of HSP70 as a molecular chaperone [46]. Indeed, locally applied heat stress during acute exercise promoted mitochondrial transcription factor A (TFAM) mRNA expression, and TFAM is involved in the coordination of mitochondrial biogenesis [35]. Accordingly, undertaking chronic exercise training with heat stress applied locally to working muscle to elevate muscle temperature above typical exercising values may promote mitochondrial biogenesis, but this has not yet been studied.

Therefore, the primary aim of the present investigation was to determine if elevating muscle temperature during exercise through the application of mild, local heat stress would promote adaptations to mitochondrial protein content in response to endurance training. We hypothesised that elevating local muscle temperature during exercise would augment training-induced increases in mitochondrial content, and that this would occur alongside greater skeletal muscle HSP70 accumulation.

## Methods

### Ethical approval

This study was performed in accordance with the standards of the Declaration of Helsinki, 2013. The Auckland University of Technology Ethics Committee approved all procedures in the human studies (21/170), and all participants provided written informed consent prior to participation. This study was not registered in a database. Data associated with this study are available from the corresponding author upon reasonable request.

### Participants

Eight recreationally-trained males regularly engaged in aerobic exercise such as running and/or cycling took part in the study (age, 30 ± 8 years; height, 182 ± 5 cm; body mass, 76.7 ± 9.6 kg; first ventilatory threshold, 198 ± 28 W; second ventilatory threshold, 240 ± 29 W; peak oxygen uptake [V̇O_2_peak], 4.1 ± 0.5 L.min^−1^). All participants were healthy, with no recent viral illness or musculoskeletal injury (< 3 months). Male and female participants were eligible to participate; however, only male participants volunteered. The achieved sample size was limited by COVID-19 restrictions. At *N* = 8, we had 50% power to detect a large magnitude effect (*d* = 0.8) with a type I error rate of 0.05 using a two-tailed test.

### Study design

**Fig. 1:**
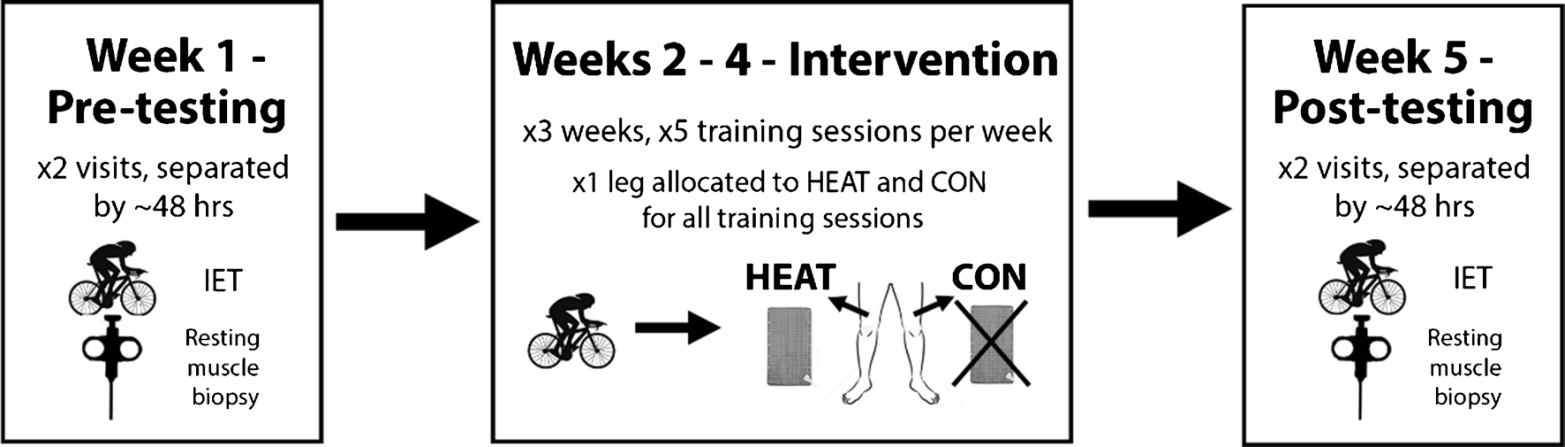
Schematic illustration of the study design. CON, control (unheated) leg; HEAT, heated leg; IET, incremental exercise test

This investigation used a longitudinal, contralateral leg design (Fig. 1). Participants initially reported to the laboratory twice during the pre-intervention week, once for an incremental exercise test to determine training zones and once for resting *vastus lateralis* microbiopsies. Subsequently, participants undertook 3 weeks of supervised exercise training in the laboratory in temperate conditions (~ 18 °C), during which mild local heat stress (~ 40 °C) was applied to the upper-leg musculature of one leg during all training sessions (HEAT), with the contralateral leg serving as the non-heated, exercising control (CON). Participants were randomised and counterbalanced to receive the local heat stress intervention to either the dominant or non-dominant leg. Following the training intervention, the pre-intervention assessments were repeated.

### Incremental exercise test

Participants initially reported to the laboratory at ~ 07:00 following a 10-h overnight fast, having refrained from vigorous exercise and alcohol for 24 h. After providing written, informed consent, height and body mass were recorded. Participants subsequently undertook an incremental cycling test on an electromagnetically braked ergometer (Excalibur Sport, Lode, Groningen, NET). A heart rate (HR) monitor was fitted for continuous collection of HR data (TICKR, Wahoo, Taiwan), and expired gases were collected throughout (TrueOne2400, ParvoMedics, Sandy, UT, USA). The test began at 95 W, with the work rate increasing by 35 W every 3 min until the respiratory exchange ratio exceeded 1.0 and attainment of the second ventilatory threshold (VT_2_) was confirmed, after which the work rate increased by 35 W every minute until task failure. The V̇O_2_peak was identified as the highest 15-s average V̇O_2_. The first ventilatory threshold (VT_1_) was identified as the V̇O_2_ at which a systematic rise in V̇E.V̇O_2_^−1^ occurred, and VT_2_ was identified as the V̇O_2_ at which a systematic rise in V̇E.V̇CO_2_^−1^ occurred. The V̇O_2_ at VT_1_ and VT_2_ were converted to power output by linear fit of the power output vs. V̇O_2_ relationship, using the last minute of V̇O_2_ data from each 3-min stage. The HR associated with VT_1_ and VT_2_ was then estimated by linear fit of the power output vs. HR relationship, using the last 30 s of HR data from each 3-min stage. The last minute of expired gas data in each 3-min stage was used to determine whole-body fat oxidation rates through standard calculations [19]. The highest observed rate of whole-body fat oxidation was accepted as the peak fat oxidation rate (PFO), as per our recent work [27].

### Resting vastus lateralis microbiopsy

Approximately 48 h following the incremental exercise test, participants returned to the laboratory at ~ 07:00 having recorded their dietary intake for 48 h, including that morning’s breakfast. A resting *vastus lateralis* sample was obtained from both legs via the microbiopsy technique. Local anaesthesia was applied to the skin and superficial muscle fascia, after which a microbiopsy needle was inserted into the mid-belly of the *vastus lateralis* to a depth of ~ 2 cm to recover ~ 20 mg of tissue using a spring-loaded mechanism (14G Ultimate Biopsy Needle, Zamar Care, Croatia). Muscle tissue was immediately frozen using dry ice, and stored at − 80 °C until further analysis. The precise location of the biopsy site was recorded relative to the line between the greater trochanter and tibial tuberosity, such that the same site could be used for the post-intervention sample.

### Training intervention

Approximately 72 h following the pre-intervention resting *vastus lateralis* microbiopsy, participants commenced a supervised, 3-week, 15-session, two-legged cycle training intervention, involving five training sessions performed each week (Table 1). During all training sessions, mild local heat stress (~ 40 °C) was applied to the upper-leg muscles of one leg (HEAT) using a wired heat pad with integrated heating element (VXHP-01, Vidawell, Auckland, NZ). The contralateral leg served as the non-heated, exercising control (CON). This method was designed to elevate the *vastus lateralis* of the heated leg ~ 2 °C above the *vastus lateralis* of the non-heated leg, whilst exposing the tissues to the same systemic environment and mechanical training stress. Participants were randomised and counterbalanced to receive local heat stress to either their dominant or non-dominant leg, which was determined prior to the first session by asking ‘If you were to shoot a ball at a target, which leg would you use to kick the ball?’ [42]. The dominant leg was considered as the kicking leg.

**Table 1:** Training intervention used in the present investigation. The five training sessions detailed below were performed each week. During all 15 training sessions, mild local heat stress (~ 40 °C) was applied to the upper-leg muscles of one leg using a wired heat pad with integrated heating element. The other leg served as the non-heated, exercising control. This training regimen was repeated three times

	Type	Session
1	Threshold	4–6 × 8 min at VT_2_ HR, 2-min recovery
2	Moderate	90 min at 95% of VT_1_ HR
3	Heavy	3 × 25 min at mid-point of VT_1_ and VT_2_ HR, 5-min recovery
4	Moderate	90 min at 95% of VT_1_ HR
5	Severe	6–10 × 3 min at ‘best effort’, 2-min recovery

*HR* heart rate, *VT*_*1*_ first ventilatory threshold, *VT*_*2*_ second ventilatory threshold

During training sessions, HR and power output were recorded continuously. Training was undertaken on participants’ own road bicycles mounted to a direct-drive indoor trainer (Kickr v5, Wahoo Fitness, Atlanta, USA). Additionally, the temperature of the *vastus lateralis* of both legs was estimated using the insulated skin temperature technique [7]. Briefly, a skin temperature thermistor was taped over the *vastus lateralis* ~ 15 cm above the patella and covered by a 6-mm-thick insulative neoprene layer. *Vastus lateralis* muscle temperature (*T*_mus_) was subsequently estimated using validated equations [7] (Eq. 1):

$$\mathrm{Estimated\;}{T}_{{\text{mus}}}\mathrm{\;at\;rest\;}=\left(0.597\times {T}_{{\text{ins}}}\right)-\left(0.439\times {T}_{{\text{ins}}}{{\text{Lag}}}_{2}\right)+\left(0.554\times {{{\text{T}}}_{{\text{ins}}}{\text{Lag}}}_{3}\right)-(0.709\times {T}_{{\text{ins}}}{{\text{Lag}}}_{4})+14.767$$

1 $$\mathrm{Estimated\;}{T}_{{\text{mus}}}\mathrm{\;during\;exercise}=\left({T}_{{\text{ins}}}\times 0.599\right)-\left[\left(0.311\times {T}_{{\text{ins}}}{\text{Lag}}4\right)+15.63\right]$$

where *T*_ins_ is insulated skin temperature over the *vastus lateralis*, *T*_ins_Lag_2_ = T_ins_ − T_ins_ two min beforehand, etc.

### Enzyme activities

For analysis of citrate synthase (CS) and complex I–V activities, ~ 10 mg of frozen muscle was cut and rinsed using cold phosphate-buffered saline (PBS) and then suspended to ~ 25 mg.mL^−1^ in PBS. Samples were then ground manually and thoroughly using a pre-cooled Dounce homogeniser. Homogenate was solubilised in extraction buffer (ab260490, Abcam®) to ~ 5 mg.mL^−1^ and incubated on ice for 20 min prior to centrifugation at 16,000 g for 10 min at 4 °C. Supernatant was extracted and stored at − 80 °C prior to further analysis. Supernatant was thawed and kinetic immunocapture assays for CS (ab119692, Abcam®; coefficient of variation [CV], 11.4%), complex I (ab109721, Abcam®; CV, 12.1%), complex II (ab228560, Abcam®, CV; 14.1%), complex III (ab287844, Abcam®; CV, 13.4%), complex IV (ab109909, Abcam®; CV, 2.3%), and complex V (ab109714, Abcam®; CV, 5.9%) activities were performed in duplicate. Enzyme activities were expressed relative to sample protein concentration using a Bradford assay (ab102535, Abcam®; CV, 6.1%). Assays were performed on a spectrophotometer (Multiskan GO, ThermoFisher Scientific Inc., Porto Salvo, POR).

### Immunoblotting

For analysis of complex I–V and HSP70 protein expression, the remaining ~ 10 mg of frozen muscle was cut and rinsed using cold PBS and then suspended in RIPA buffer (20 µL_._mg^−1^ muscle) made with protease inhibitor cocktail (ab65621, Abcam®, 20 μL_._mL^−1^) and PMSF (ab141032, Abcam®, 5 μL_._mL^−1^), and PBS (20 μL_._mg^−1^). Samples were then ground manually and thoroughly using a pre-cooled Dounce homogeniser. Homogenate was extracted and incubated on ice for 20 min prior to centrifugation at 16,000 g for 10 min at 4 °C. Supernatants was extracted and stored at − 80 °C prior to further analysis.

Total protein concentration was determined using a BCA-protein kit (Pierce BCA protein assay Kit, Thermo Fisher Scientific) with bovine serum albumin (BSA) as a standard. Sample aliquots containing ~ 5 µg protein suspended in 1 × Laemmli buffer (0.5 M Tris/HCl, pH 6.8, 800 mM 2-mercaptoethanol, 2 mM EGTA, 10% glycerol, 2% SDS, 0.25% Bromophenol Blue) were resolved by SDS-PAGE using 12.5% hand cast gels and transferred to PVDF membranes (Trans-Blot Turbo transfer pack, Bio-Rad Laboratories) using a Trans-Blot Turbo transfer system (Bio-Rad Laboratories). Membranes were blocked using 2% fish gelatine in Tris buffered saline-0.1% Tween-20 (TBST) for 2 h, then washed in TBST for 15 min, and incubated overnight at 4 °C in primary antibodies diluted in TBST with 3% w/v BSA. Membranes were probed for HSP70 (1:500, H5147, Sigma-Aldrich) and the five oxidative phosphorylation complex subunits using an antibody cocktail (1:2000, ab110411, Abcam). After the removal of primary antibody, the membranes were washed in TBST for 15 min and then incubated for 2 h in horseradish peroxidase-conjugated secondary antibody diluted in TBST with 2% fish gelatine. A ChemiDoc MP Imaging System (Bio-Rad Laboratories) was used to capture the images in the presence of chemiluminescent substrate (Clarity Western Substrate; Bio-Rad Laboratories). Bands were quantified using ImageLab 6.1 software (Bio-Rad Laboratories). To adjust for protein loading, bands of interest were standardised by probing for glyceraldehyde 3-phosphate dehydrogenase (GAPDH; 1:5000, ab9485, Abcam). To control for variation between gels, a pooled sample was loaded in every gel and all target proteins are quantified relative to the pooled sample.

### Statistical analysis

Data is expressed as mean ± standard deviation. Statistical analysis was performed with GraphPad Prism Version 9.3.1 (GraphPad Software, San Diego, CA, USA). Normality of datasets was assessed using the Shapiro–Wilk test. Pre- and post-intervention V̇O_2_peak, VT_1_, VT_2_, and PFO were compared using paired *t*-tests. The effect of local heat stress application during the first of each training session type on estimated *T*_mus_ was assessed using a two-way analysis of variance with repeated measures. The effect of local heat stress application during training on skeletal muscle variables was assessed using two-way analyses of variance with repeated measures. Training-induced changes in skeletal muscle variables are expressed as fold changes and compared between legs using two-tailed paired *t*-tests (or non-parametric equivalents). Significance was accepted when *P* ≤ 0.05.

## Results

**Fig. 2:**
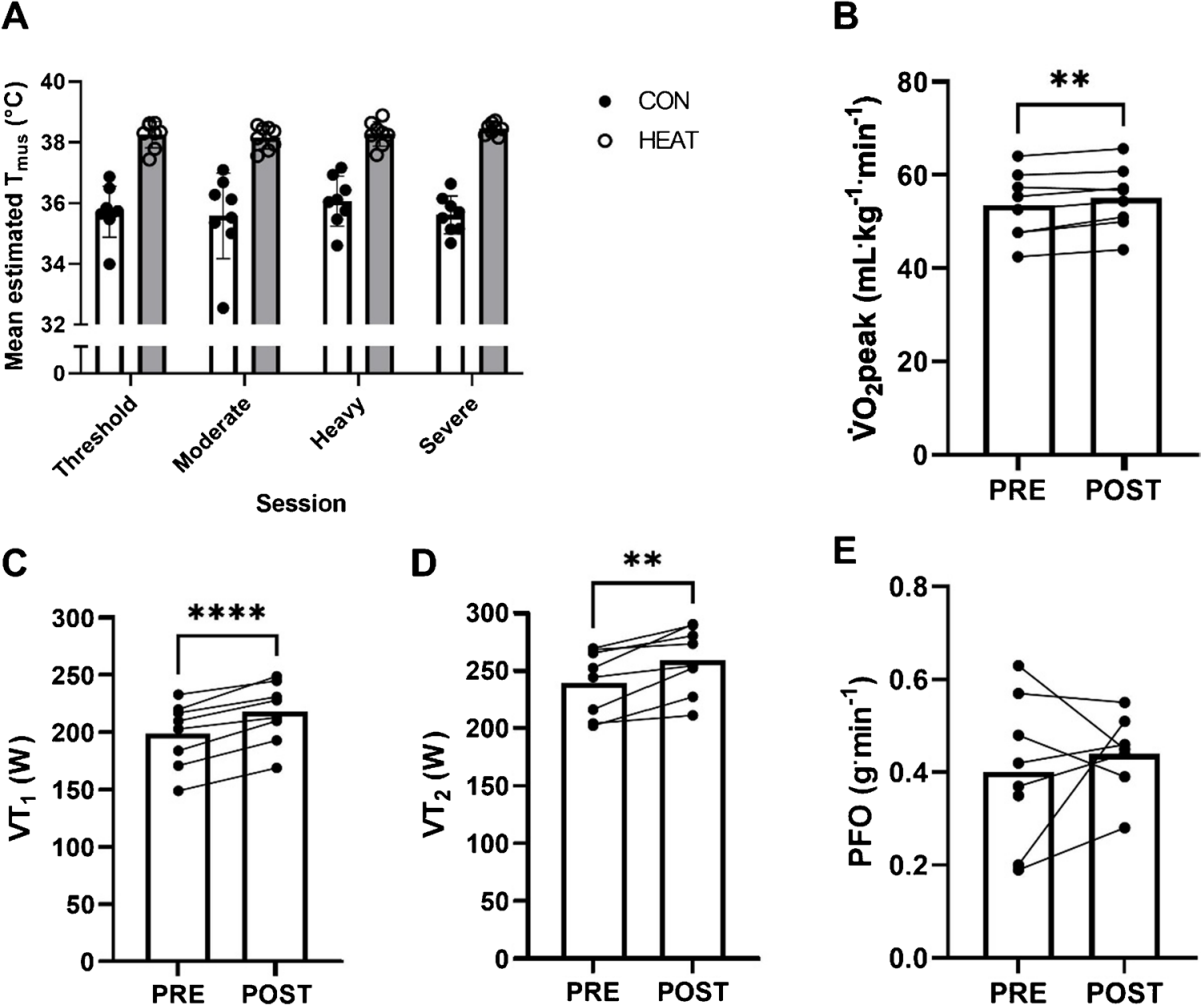
Verification of the effectiveness of the local heat stress and training intervention. **a** Mean estimated *vastus lateralis* temperature (*T*_mus_) in the control (CON) and heated (HEAT) leg during the first threshold, moderate, heavy, and severe-intensity training session. **b** Peak oxygen uptake (V̇O_2_peak), **c** first ventilatory threshold (VT_1_), **d** second ventilatory threshold (VT_2_), and **e** peak fat oxidation rate (PFO) before (PRE, week 1) and after (POST, week 5) the training intervention. One post-intervention datapoint for PFO was missed due to a technical error. The symbol ‘**’ indicates *P* < 0.01, and the symbol ‘****’ indicates *P* < 0.0001

Mean estimated *T*_mus_ of CON and HEAT was calculated for the first of each session type (Fig. 2a). A main effect of local heat stress application was observed, whereby mean estimated *T*_mus_ was greater in HEAT than CON (38.3 ± 0.4 vs. 35.7 ± 0.9 °C, mean difference 2.5 ± 0.8 °C, *P* < 0.0001), with no main effect of session type (*P* = 0.562) or interaction between local heat stress application and session type (*P* = 0.148). Increased V̇O_2_peak (53 ± 7 vs. 55 ± 7 mL^.^kg^−1^_._min^−1^, *P* = 0.01, Fig. 2b), VT_1_ (192 ± 28 vs. 217 ± 27 W, *P* = 0.0001, Fig. 2c), and VT_2_ (240 ± 29 vs. 260 ± 29 W, *P* = 0.003, Fig. 2d) was observed post-intervention, with no effect on PFO (0.40 ± 0.16 vs. 0.44 ± 0.09 g_._min^−1^, *P* = 0.615, Fig. 2e). Body mass was unchanged post-intervention (76.7 ± 9.6 vs. 76.4 ± 9.2 kg, *P* = 0.507).

**Fig. 3:**
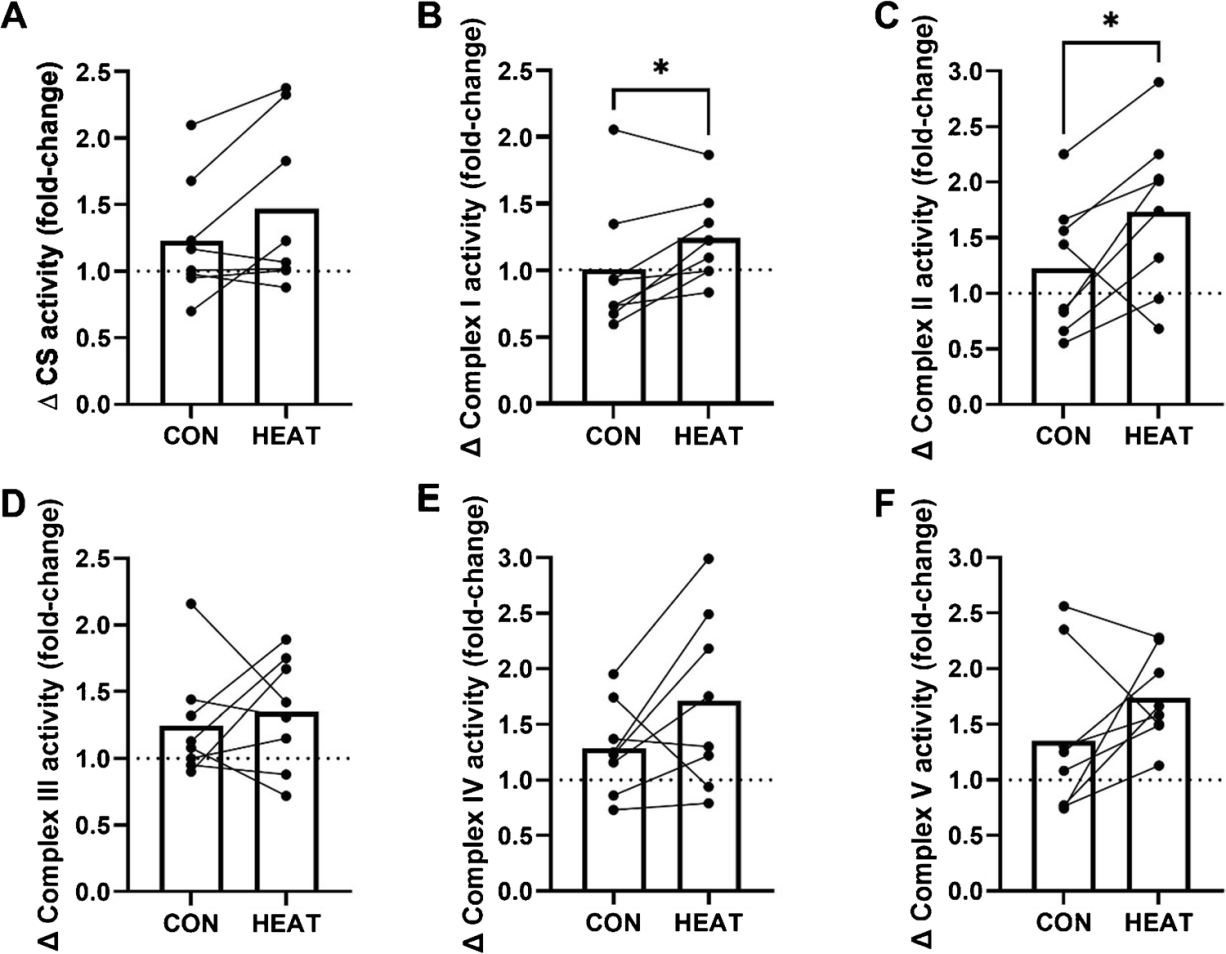
Mitochondrial enzyme activity adaptations to three weeks of bilateral cycling training in the *vastus lateralis* of a control leg (CON) and a leg exposed to mild, local heat stress during all training sessions (HEAT). **a** Citrate synthase, **b** complex I, **c** complex II, **d** complex III, **e** complex IV, **f** complex V. Bars indicate means and dots indicate individual values, linked for each participant. The symbol ‘*’ denotes *P* ≤ 0.05

**Fig. 4:**
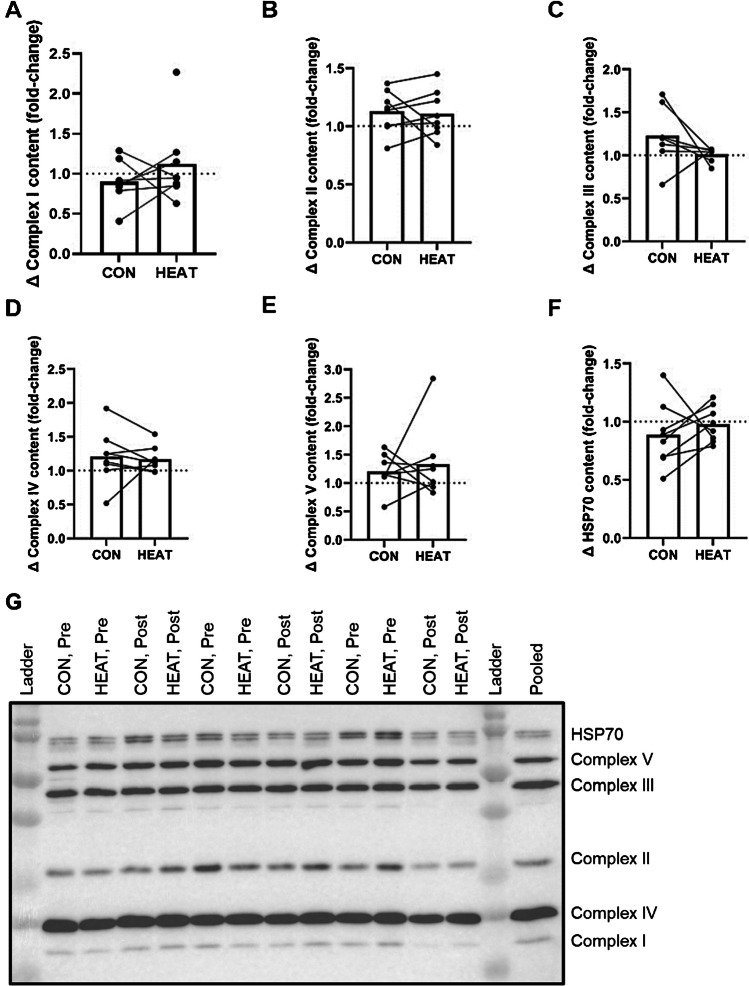
Mitochondrial protein expression adaptations to three weeks of bilateral cycling training in the *vastus lateralis* of a control leg (CON) and a leg exposed to mild, local heat stress during all training sessions (HEAT). **a** complex I, **b** complex II, **c** complex III, **d** complex IV, **e** complex V, and **f** heat shock protein 70 (HSP70). Bars indicate means and dots indicate individual values, linked for each participant. **g** Representative immunoblot

No significant between-limb differences in enzyme activity were observed pre-intervention (in all cases, *P* > 0.100). An interaction between local heat stress application and training was not observed for CS activity (*P* = 0.059). The training-induced increase in CS activity was not significantly larger in HEAT than CON (fold-change, 1.47 ± 0.62 vs. 1.23 ± 0.45, *P* = 0.068, Fig. 3a). An interaction between local heat stress application and training for complex I activity was observed (*P* = 0.012). The training-induced increase in complex I activity was larger in HEAT than CON (fold-change, 1.24 ± 0.33 vs. 1.01 ± 0.49, *P* = 0.029, Fig. 3b). An interaction between local heat stress application and training was not observed for complex II activity (*P* = 0.222). However, the training-induced increase in complex II activity was greater in HEAT vs. CON (fold-change, 1.73 ± 0.73 vs. 1.23 ± 0.59, *P* = 0.042, Fig. 3c). Interactions between local heat stress application and training, and differences in training-induced increases between CON and HEAT, were not observed for complex III, IV, or V activity (Fig. 3d–f). No significant effects of training, or interactions between local heat stress application and training, were observed for complex I–V or HSP70 protein expressions (Fig. 4).

## Discussion

The primary aim of the present investigation was to determine the effect of elevating muscle temperature during exercise through application of mild, local heat stress on adaptations to mitochondrial protein content in response to endurance training. Our main observations were that the application of mild, local heat stress during cycling training: (i) elicited significantly greater adaptations to complex I and II activities, but not complex III-V or citrate synthase activity, (ii) did not impact mitochondrial protein expression, and (iii) did not impact HSP70 expression. Our data therefore provide partial support for our hypothesis that elevating muscle temperature during exercise augments training-induced adaptations to mitochondrial enzyme activity, although more work is necessary to explore this effect.

The effectiveness of our experimental model for studying the impact of elevating muscle temperature during exercise on mitochondrial adaptations is evidenced by improvements in physiological parameters relevant to endurance, and the consistently greater estimated *T*_mus_ in the heated compared to control limb (Fig. 2). Specifically, VT_1_ (10 ± 4%), VT_2_ (8 ± 6%), and V̇O_2_peak (3 ± 2%) significantly increased from pre-to-post training, which are evidence that our training programme was effective at stimulating endurance-related adaptations [27, 29, 31], and therefore allowed us to delineate the effect of local heat application on mitochondrial biogenesis. Whilst we cannot ascribe these improvements solely to mitochondrial factors given the myriad adaptations that occur in response to endurance exercise training, the timeframe of the intervention in the present study [28, 39] has been adequate to increase mitochondrial enzyme activity and protein expression in previous studies [4, 20, 28, 34]. The ~ 2 °C between-limb difference in estimated *T*_mus_ is similar to that which we observed between 20 min of moderate-intensity exercise in 18 vs. 40 °C [26], and to a study of 40 min of moderate-intensity exercise in 20 vs. 40 °C measuring muscle temperature directly [6], although lower than studies observing mitochondrial biogenesis following passively applied local heat stress using short-wave diathermy (~ 3.9 °C) [10]. Studies of passive exposure are an inherently different model, as the control limb remains at resting temperatures; muscle temperature does rise above resting temperatures during exercise in temperate conditions [6]. As the estimated muscle temperature technique has not been validated with concomitant local heat stress application, direct assessment of indwelling skeletal muscle temperature would have strengthened the present work. However, we are confident that the intervention sufficiently manipulated working skeletal muscle temperature to allow us to test our hypothesis.

Our data provides partial support for the hypothesis that elevating muscle temperature during exercise augments adaptations to mitochondrial enzyme activity in response to endurance training. Specifically, we report greater training-induced increases in complex I and II enzyme activities in HEAT vs. TEMP (Fig. 3bc). Increases in mitochondrial enzyme activities following training interventions have typically been interpreted as evidence of mitochondrial biogenesis [2], given previously-observed relationships with total mitochondrial content measured using transmission electron microscopy [22]. The absence of significant effects on citrate synthase activity, or in complex III-V activities, may be related to normal variability in the assays themselves (CV, 2.3–13.4%), small differences in the location of pre- and post-training biopsy sites [16], and the small sample size. We mitigated the effect of these error sources by measuring the pre-training biopsy sites for replication post-training, and by use of the highly-controlled contralateral leg design. It remains possible that real effects were missed due to having small magnitudes relative to the typical variability in the measurements, or that real effects were only observed for complex I and II, which emphasises the need for further studies with greater statistical power. Overall, our data provides some initial suggestion that elevated muscle temperature during exercise may positively affect some mitochondrial enzyme activity adaptations, although more work is needed to elucidate this effect.

Opposed to this conclusion is our mitochondrial protein expression data, derived from immunoblotting (Fig. 4). We observed no significant main effects of training on complex I–V expression, or interactions between training and application of mild, local heat stress. When considering the individual datasets, the only significant effect of training was an increase in complex IV protein expression observed in HEAT (fold-change, 1.17 ± 0.19, *P* = 0.013). Why increased mitochondrial enzyme activity would be observed without concomitant increases in mitochondrial protein expression is not clear and may relate to technical challenges and variability associated with immunoblotting [1], alongside the methodological aspects cited above. It is also possible that mitochondrial enzyme activity was enhanced independently of enzyme content, perhaps due to adaptations to co-factors. Regardless, whilst not conclusive, our data supports further research in larger studies with measures of mitochondrial content and respiratory activity to assess if elevating muscle temperature during exercise promotes mitochondrial adaptation and is therefore a driver of mitochondrial biogenesis.

Contrary to our hypothesis, application of mild, local heat stress during endurance training did not promote a detectable increase in HSP70 protein expression (Fig. 4f). In fact, a main effect of training on HSP70 protein expression was not observed. Previous work has reported increased HSP70 expression following endurance training [24, 25], although this has not always been observed [33]. We hypothesised that greater increases in HSP70 protein expression would be observed following HEAT based on recent work with passive heat stress using short-wave diathermy [9, 10], and that greater HSP70 accumulation would contribute to greater mitochondrial adaptations in HEAT [13, 46]. It is possible the training programme used in the present study, along with the magnitude of the thermal stress in HEAT, was insufficient to stimulate HSP70 accumulation. It is also possible the timing of post-exercise biopsies (~ 4–6 days following the last intervention session) meant changes to HSP70 expression were missed. Nevertheless, our data do not support the hypothesis that elevated muscle temperature during exercise training promotes HSP70 accumulation, although more work is warranted.

The mechanism behind any effect of elevated muscle temperature during exercise on adaptations to mitochondrial enzyme activity is not clear but could plausibly be related to greater acute increases in AMPK activation in response to individual training sessions in HEAT, via greater muscle glycogenolysis. Exercise with elevated skeletal muscle temperature has been shown to increase muscle glycogen utilisation [5, 40], and glycogen-AMPK binding inhibits AMPK activity [30]. Therefore, greater muscle glycogen utilisation in a limb exposed to mild, local heat stress during training might be expected to increase AMPK activation. This contention is supported by observations of an inverse relationship between muscle glycogen content and AMPK activity [37, 38] and observed positive effects of low glycogen exercise on AMPK activity [43]. Greater AMPK activation is thought to induce PGC-1α phosphorylation [18], a key regulator of mitochondrial biogenesis [11, 45]. As the purpose of the present study was to assess how application of local heat stress during exercise impacts training-induced adaptations to mitochondrial enzyme activity and protein expression, we obtained resting muscle samples before and after the intervention period, with the post-intervention samples obtained several days following the last intervention session. Given the time-course of signalling responses to exercise [21], our study design is not appropriate for assessing signalling-related responses to exercise performed with application of local heat stress. Accordingly, further research into the effect of elevating muscle temperature during exercise on signalling responses, including AMPK activation, is warranted.

In conclusion, this study suggests that elevated muscle temperature during exercise might enhance certain mitochondrial enzyme activities (complex I and II). However, further research is required to fully understand this effect due to variations in mitochondrial enzyme activity responses and the absence of changes in mitochondrial protein expression. We consider these findings as a foundation for conducting more comprehensive investigations into this hypothesis, involving larger-scale studies with extended exercise training durations.

## Data Availability

Data is available from the corresponding author upon reasonable request.

## Abbreviations

AMP: Adenosine monophosphate
AMPK: 5′AMP-activated protein kinase
CON: Control leg
CS: Citrate synthase
GAPDH: Glyceraldehyde 3-phosphate dehydrogenase
HEAT: Heated leg
HR: Heart rate
HSP70: Heat shock protein 70
PFO: Peak fat oxidation rate
*T*_mus_: Muscle temperature
V̇CO_2_: Rate of carbon dioxide production
V̇O_2_: Rate of oxygen consumption
V̇O_2_peak: Peak rate of oxygen consumption
VT_1_: First ventilatory threshold
VT_2_: Second ventilatory threshold

## Code availability

None used.
